# NG-Tax 2.0: A Semantic Framework for High-Throughput Amplicon Analysis

**DOI:** 10.3389/fgene.2019.01366

**Published:** 2020-01-23

**Authors:** Wasin Poncheewin, Gerben D. A. Hermes, Jesse C. J. van Dam, Jasper J. Koehorst, Hauke Smidt, Peter J. Schaap

**Affiliations:** ^1^Laboratory of Systems and Synthetic Biology, Wageningen University & Research, Wageningen, Netherlands; ^2^Laboratory of Microbiology, Wageningen University & Research, Wageningen, Netherlands

**Keywords:** operational taxonomic unit, amplicon sequence variants, taxonomic classification, FAIR, semantic web, RDF, ontology, SPARQL

## Abstract

NG-Tax 2.0 is a semantic framework for FAIR high-throughput analysis and classification of marker gene amplicon sequences including bacterial and archaeal 16S ribosomal RNA (rRNA), eukaryotic 18S rRNA and ribosomal intergenic transcribed spacer sequences. It can directly use single or merged reads, paired-end reads and unmerged paired-end reads from long range fragments as input to generate *de novo* amplicon sequence variants (ASV). Using the RDF data model, ASV’s can be automatically stored in a graph database as objects that link ASV sequences with the full data-wise and element-wise provenance, thereby achieving the level of interoperability required to utilize such data to its full potential. The graph database can be directly queried, allowing for comparative analyses of over thousands of samples and is connected with an interactive Rshiny toolbox for analysis and visualization of (meta) data. Additionally, NG-Tax 2.0 exports an extended BIOM 1.0 (JSON) file as starting point for further analyses by other means. The extended BIOM file contains new attribute types to include information about the command arguments used, the sequences of the ASVs formed, classification confidence scores and is backwards compatible. The performance of NG-Tax 2.0 was compared with DADA2, using the plugin in the QIIME 2 analysis pipeline. Fourteen 16S rRNA gene amplicon mock community samples were obtained from the literature and evaluated. Precision of NG-Tax 2.0 was significantly higher with an average of 0.95 vs 0.58 for QIIME2-DADA2 while recall was comparable with an average of 0.85 and 0.77, respectively. NG-Tax 2.0 is written in Java. The code, the ontology, a Galaxy platform implementation, the analysis toolbox, tutorials and example SPARQL queries are freely available at http://wurssb.gitlab.io/ngtax under the MIT License.

## Introduction

High-throughput sequencing technologies have empowered our ability to study complex environmental and host-associated microbial communities. Of these technologies, amplicon sequencing targeting marker genes is currently the most cost-effective tool to assess the microbial composition of large numbers of samples (Tringe and Rubin, 2005; Yarza et al., 2014; Stulberg et al., 2016). By using smart multiplexing techniques hundreds of samples can be sequenced at once while sequencing costs per sample are further reduced leading to immense amounts of microbial community composition data available for large scale comparisons.

High-throughput amplicon sequencing is, however, inevitably noisy. Due to PCR artefacts and low-quality base calls, a fraction of the amplicon reads will contain one or more sequence errors (error-reads), which in turn could lead to false taxonomic inferences. One strategy to reduce the number of false taxonomic inferences due to these error-reads, is to cluster amplicon reads by sequence identity in operational taxonomic units (a process called OTU-picking) at some user defined identity thresholds. To build these OTUs, centroid or seed sequence-based greedy clustering approaches are frequently used (Stackebrandt and Goebel, 1994; Konstantinidis and Tiedje, 2005; Godzik and Li, 2006; Edgar, 2010). Centroid based OTU-picking approaches however, have a number of disadvantages as they require a predefined identity threshold, while the representative centroid sequence is influenced by selection of the seed, sequence input order and the amount of amplicon sequences and PCR error present in the sample, all of which make OTU-picking by clustering sample dependent and therefore, in principle, not suitable for comparisons between different sets of samples (Callahan et al., 2016). Recent studies have shown that a *de novo* clustering approach using exact matches would yield better results (Ramiro-Garcia et al., 2016; Callahan et al., 2017). These exact match sequence clusters have been termed Amplicon Sequence Variants (ASVs), sub-OTUs or zero-radius OTUs (Tikhonov et al., 2015; Callahan et al., 2016; Edgar, 2018). The rationale is that an ASV is not a representative sequence from a cluster of similar sequences, but is directly derived from a biological entity. An ASV can be separated from error-reads on the basis of the expectation that due to the biological origin, a real sequence variant is located at a fixed position in the amplicon sequence and therefore more likely to be repeatedly observed in those samples where the particular biological variant is present. Error-reads are assumed to be present at a relatively low abundance, and because sequence errors are also positionally dispersed (Schirmer et al., 2016) they are unable to form meaningful exact match ASV clusters. In NG-Tax, an exact match OTU-picking algorithm is used to find ASV forward and reverse sequence read pairs. Likely erroneous ASVs are rejected if their read count does not exceed an experimentally defined dynamic threshold that takes the evenness of the distribution into account (Ramiro-Garcia et al., 2016). In the past the accuracy of NG-Tax has been benchmarked against QIIME (Caporaso et al., 2010), using synthetic mock communities and has been shown to outperform it (Ramiro-Garcia et al., 2016).

Unlike centroid based OTUs which work with representative sequences, ASV sequences are believed to directly descend from an existing biological entity, and the presence of this entity can therefore be validly compared across many samples (Callahan et al., 2017). Such large-scale analyses would require tracking of multiple ASVs over multiple samples and thus a high degree of interoperability. Proper data handling can be achieved through the application of the FAIR data principles which are intended to make the information Findable, Accessible, Interoperable and Reusable (Wilkinson et al., 2016). We adopted these principles in NG-Tax 2.0 through implementation of a semantic framework using a Linked Data format (RDF) for data serialization and handling, combined with a strictly applied ontology. In NG-Tax 2.0 ASV amplicon sequences are automatically converted into a semantic data model, ASV objects, that link ASV sequences with the full data-wise and element-wise provenance thereby achieving the level of interoperability required to utilize such data to its full potential.

NG-Tax 2.0 is a complete redesign and rewrite of the NG-Tax amplicon analysis pipeline. In NG-Tax 2.0 many of the limitations of NG-Tax have been addressed and as a result NG-Tax 2.0 has evolved into a highly automated framework for high-throughput classification and comparative analysis of marker gene amplicon sequences.

Using ten mock communities publicly available from the Mockrobiota database (Bokulich et al., 2016) and data from four staggered mocks described by (Tourlousse et al., 2017) the precision and recall of NG-Tax 2.0 was evaluated against DADA2 (Callahan et al., 2016) using the plugin in the QIIME 2 pipeline. The known relative abundance of each ASV in these mock communities enabled a precise evaluation of the tools on how they perform in predicting the number of species, their relative abundance and their taxonomic classification. The integrative power of using a semantic framework is demonstrated by performing a meta-analysis across the mock samples and multiple reference databases.

## Materials and Methods

### NG-Tax 2.0

NG-Tax 2.0 is written in Java with Gradle as build system. A Galaxy web implementation (Afgan et al., 2016) is also available. A k-bounded Levenshstein distance function (Hawkins et al., 2018) was implemented in Java to measure the edit distances between amplicon sequences in OTU-picking and between ASV sequences and reference database sequences for taxonomic annotation of ASV objects. The distance function was slightly modified to account for phantom out of word frame insertion and deletions.

### NG-Tax 2.0 Semantic Framework

An NG-Tax 2.0 specific expansion of the GBOL ontology (van Dam et al., 2019) was developed in Protégé (Musen and Team, 2015). Empusa (van Dam et al., 2019) was used to convert the ontology to a Java API. As a result, picked ASVs, taxonomic inferences and linked metadata can be automatically stored in a graph database and can be directly retrieved and compared through a list of (routine) SPARQL queries. A list of routine SPARQL queries is provided (**Data Sheet 1**), the output of which directly interacts with the NG-Tax 2.0 data analysis and visualization toolbox that is based on Rshiny (Chang et al., 2017). RDF (turtle) files were imported into a local GraphDB (http://graphdb.ontotext.com/) repository and queried using the SPARQL query language.

### Mock Communities

Mock communities were retrieved from the Mockrobiota project (Bokulich et al., 2016). Ten demultiplexed 16S-rRNA gene mock communities were obtained (**Table 1**).

**Table 1:** Mock communities used for NG-Tax 2.0 benchmarking.

Mockrobiota #	Composition	Read length	Reference
Mock13	21 bacterial strains, evenly distributed	250/250	Kozich et al., 2013
Mock14	21 bacterial strains, evenly distributed	250/250	
Mock15	21 bacterial strains, evenly distributed	250/250	
Mock16	49 bacterial strains, 10 archaea, evenly distributed	250/250	Schirmer et al., 2015
Mock18	15 bacterial strains, evenly distributed	250/250	Tourlousse et al., 2017
Mock19	15 bacterial strains, 12 synthetic spike-in standards, evenly distributed	250/250	
Mock20	20 bacterial strains, evenly distributed	301/301	Gohl et al., 2016
Mock21	20 bacterial strains, staggered	301/301	
Mock22	20 bacterial strains, evenly distributed	301/301	
Mock23	20 bacterial strains, staggered	301/301	
SRX1868061- SRX1868064	15 bacterial strains, 12 synthetic spike-in standards, staggered	250/250	Tourlousse et al., 2017

*All mocks utilized the V4 region.

### Bioinformatic Analysis

#### General

The mock communities were analysed using: NG-Tax 2.0 and QIIME2 using the DADA2 plugin (Hall and Beiko, 2018, p. 2). Apart from the variation in amplicon read length, all settings remained as the default. The SILVA reference database was used for the taxonomic classification (Yilmaz et al., 2014). For comparison purposes, three incremental stable versions of the database were downloaded from https://www.arb-silva.de/download/archive/being: 123, 128 and 132 (latest). Additionally, a custom 16S rRNA gene database was created *de novo* using sequences from (Hug et al., 2016; **Data Sheet 2**) as input. For comparison the description line of the sequences was converted to contain the taxonomic lineage in the SILVA format. The chimera detection process has been described by Ramiro-Garcia et al., 2016. Briefly, chimeras are detected using the following condition: if the forward and reverse read of the ASV are identical to two different ASVs in the same sample and the abundance of the matched ASVs are at least twice of the abundance, then the ASV is marked as chimeric.

#### Lookup Table

For taxonomic annotation of ASV objects, NG-Tax 2.0 creates a lookup table from reference sequences. There are two options.

When a multiple alignment file, such as the 50,000 columns long SILVA alignment is provided, NG-Tax 2.0 assumes that the sequence of the primer region is conserved in the alignment. Using a regular expression which takes care of IUPAC wildcard characters, NG-Tax 2.0 finds in each aligned sequence, primer start and stop positions, starting with the first aligned sequence and keeps on doing this until a consensus start and stop column position is obtained (defined as: the start and the stop position of the primer are found to occur in the same columns/positions a 1,000 times). It then assumes that the region of interest is in the columns between the primer columns, extracts this region, removes alignment gaps, trims the sequences to the chosen forward and reverse read length and subsequently transforms the sequences into a four-column lookup table. An example is shown in **Table 2**.

**Table 2:** Example of the look-up table.

AGGAT…	CGACA…	Bacteria;Bacteroidetes;Flavobacteriia;Flavobacteriales;Flavobacteriaceae;Chryseobacterium	148
AGGAT…	CGACA…	Bacteria;Proteobacteria;Alphaproteobacteria;Rickettsiales;Anaplasmataceae;Wolbachia	276
GGGAT…	CGACA…	Bacteria;Cyanobacteria;Chloroplast;Corchorus_capsularis;_;_	3
GGGAT…	CGACA…	Bacteria;Cyanobacteria;Chloroplast;Isatis_tinctoria;_;_	4
GGGAT…	CGACA…	Bacteria;Cyanobacteria;Chloroplast;Aethionema_carneum;_;_	1

For special cases such when strain specific markers have been developed, or for studying a new species or a designed community in a closed system, NG-Tax 2.0 can also build a custom lookup table from unaligned reference sequences. For this NG-Tax 2.0 uses a regular expression representing the (degenerate) primers used in amplification to find the region of interest, taking into account a single mismatch with the exception of the most 3-prime nucleotide of the primer which must either have a perfect match or a G/T mismatch for amplification to occur. The sequence region in between the primers is subsequently used to build the lookup table as described above. To illustrate this approach a custom 16S rRNA gene database was created *de novo* using sequences from (Hug et al., 2016, **Data Sheet 2**) as input.

#### NG-Tax 2.0 Configuration

To use the NG-Tax 2.0 command line interface, users need to provide the paired-end amplicon reads in comma separated format (-fS), the mapping file (-mapFile), a reference database such as the SILVA database (-refdb) for creation of the look-up table, the selected forward and the reverse primer used for selection of the amplified region in the reference file (-for_p and -rev_p), the name for the output RDF file (-t), and the name for the output BIOM file (-b), and they need to specify whether the primers were already removed from the sequences or not (-primerRemoved). Various amplicon read lengths were used in the analysis: 70, 100, 140, 200 and 240 nt. Other settings were kept as the default as the following: -minPerT 0.1, -identLvl 100, -errorCorr 1 and -classifyRatio 0.8. as described in (Ramiro-Garcia et al., 2016). A full list of options can be found at http://wurssb.gitlab.io/ngtax/commandLine.html. Parameters are stored in the output file in “args” section of the extended BIOM and RDF file.

#### QIIME2-DADA2 Configuration

For QIIME2, the latest SILVA database for QIIME2 (version 132) was downloaded from the official QIIME2 website at https://docs.qiime2.org/2018.11/. SILVA database version 128 was downloadable through the forum page (https://forum.qiime2.org/t/silva-128-classifiers-available-for-download/3558). Silva database version 123 needed to be created manually through q2-feature-classifier tutorial https://docs.qiime2.org/2018.6/tutorials/feature-classifier/).

To analyse the data with this pipeline, we imported reads into QIIME2 as an artefact using the Casava 1.8 paired-end demultiplexed Fastq format. DADA2 (Callahan et al., 2016) was selected as the method for quality control using the following parameters: –p-trim-left-f 19 and –p-trim-left-r 20 as the length of the primer combined with various read lengths, 140, 150, 180, 200, 220 and 240, for both –p-trunc-len-f and –p-trunc-len-r. The trimming option (–p-trim-left-f and –p-trim-left-r) was used only for mock 16, 18, 19, 22 and 23. This results in a feature table, representative sequences and a statistical outcome captured during this denoising step. Next, classify-sklearn was used to classify the taxonomic lineage of the representative sequences based on the given database. Then, the classified sequences were collapsed with the feature table in order to produce an OTU table at a certain taxonomic lineage resolution based on the user input, such as 6 for genus level. Finally, the OTU table is exported into a Hierarchical Data Format (HDF5) file format which can be converted in to a tab separated values (tsv) or a JavaScript object notation (json) file format using the BIOM package (http://biom-format.org/documentation/biom_conversion.html#general-usage-examples).

### Statistics

#### Binary Classifier

Comparison between the expected and the predicted results using the confusion matrix.

$$\mathrm{recall}=\frac{\mathrm{TP}}{\mathrm{TP}+\mathrm{FN}}$$

$$\mathrm{precision}=\frac{\mathrm{TP}}{\mathrm{TP}+\mathrm{FP}}$$

$$F-\mathrm{score}=2\times \frac{\mathrm{precision}\times \mathrm{recall}}{\mathrm{precision}+\mathrm{recall}}$$

where TP is the number of true positives, FN is the number of false negatives and FP is the number of false positives. A TP was defined as an exact match at genus level.

#### Modified Rv Coefficient

Comparison between two weighted adjacency matrices, which in this case is the microbial composition and their relative abundance. The results can be interpreted as Pearson’s correlations.

$$RV_{2}\left(X,Y\right)=\frac{\mathrm{Vec}{\left(\tilde{XX^{\prime }}\right)}^{\prime }\mathrm{Vec}\left(\tilde{YY^{\prime }}\right)}{\sqrt{\mathrm{Vec}{\left(\tilde{XX^{\prime }}\right)}^{\prime }\mathrm{Vec}\left(\tilde{XX^{\prime }}\right)\times \mathrm{Vec}{\left(\tilde{YY^{\prime }}\right)}^{\prime }Vec\left(\tilde{YY^{\prime }}\right)}}$$

Given matrix $\tilde{XX^{\prime }}=\left[XX^{\prime }-diag\left(XX^{\prime }\right)\right]$, where *diag*(*XX*′) is a matrix containing only the diagonal elements of *XX*′ on its diagonal, and zero’s elsewhere. The same definition also applied to *YY*′.

## Results

**Figure 1:**
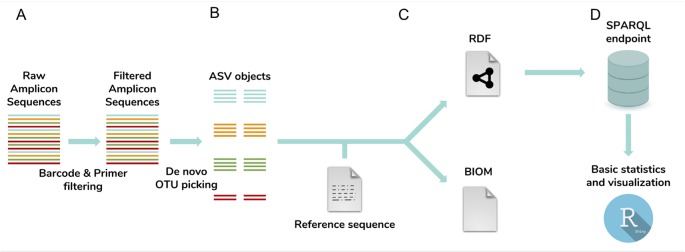
NG-Tax 2.0 workflow. The workflow consists of four main steps: **(A)** barcode and primer filtering, **(B)** *de novo* OTU-picking of ASV sequences, artefact filtering, correction for the impact of error reads on ASV relative abundance estimates and taxonomic inference; **(C)** ASV object serialization and storage. ASV sequences, taxonomic inferences and data provenance including library and sample names and used settings are exported and stored as ASV objects in an RDF triple store graph database and optionally exported in the Biom 1.0 file format. **(D)** Downstream analysis tool box. ASV data and meta-data can be directly queried and analysed through the SPARQL endpoint. The Rshiny toolbox directly provides standard statistics and visualizations using predefined SPARQL queries.

NG-Tax 2.0 is fully written in Java and can be executed from the command line, while a Galaxy toolbox implementation (Afgan et al., 2016) is also available. Using multiplexed amplicon sequences as input, NG-Tax 2.0 executes four major tasks: demultiplexing and amplicon read cleaning, generation of ASV objects (a process generally referred to as OTU-picking), denoising and taxonomic assignment. Processed samples, derived ASV sequences, taxonomic inferences and data provenance are automatically linked and serialized in an RDF-triple store format and can be exported as an extended Biom 1.0 file for compatibility reasons (**Figure 1**).

### Development of the Semantic Framework

NG-Tax 2.0 uses the RDF data model to capture and store analysis results and associated data provenance as ASV objects. To ensure consistency and to have a high degree of interoperability and reusability a strictly defined ontology was created, focusing on its function as file format and as database schema. The modular design ensures that the ontology can be extended and currently consists of eight main classes (**Table 3**).

**Table 3:** Description of the NG-Tax 2.0 ontology main classes.

Main ontology class	Description
Library*	Description of samples in a library
Sample	Description of PCRPrimers, BarcodeSet and ASVSet
Sequence	ASVSequence: ASV forward and reverse sequences
SequenceSet*	ASVSet, RejectedAsChimera, RejectedASV BarcodeSet, PCRPrimerSet
Taxon	Taxon name and rank annotation of an ASVSet
ASVAssignment	Taxon information and related provenance
Provenance	Interlinks ProvenanceClassification, containing tool specific information with the input Library
ProvenanceClassification*	Contains confidence score of taxonomic assignment and user input command argument in the analysis

*NG-Tax 2.0 specific extensions of the gbol ontology.

**Figure 2:**
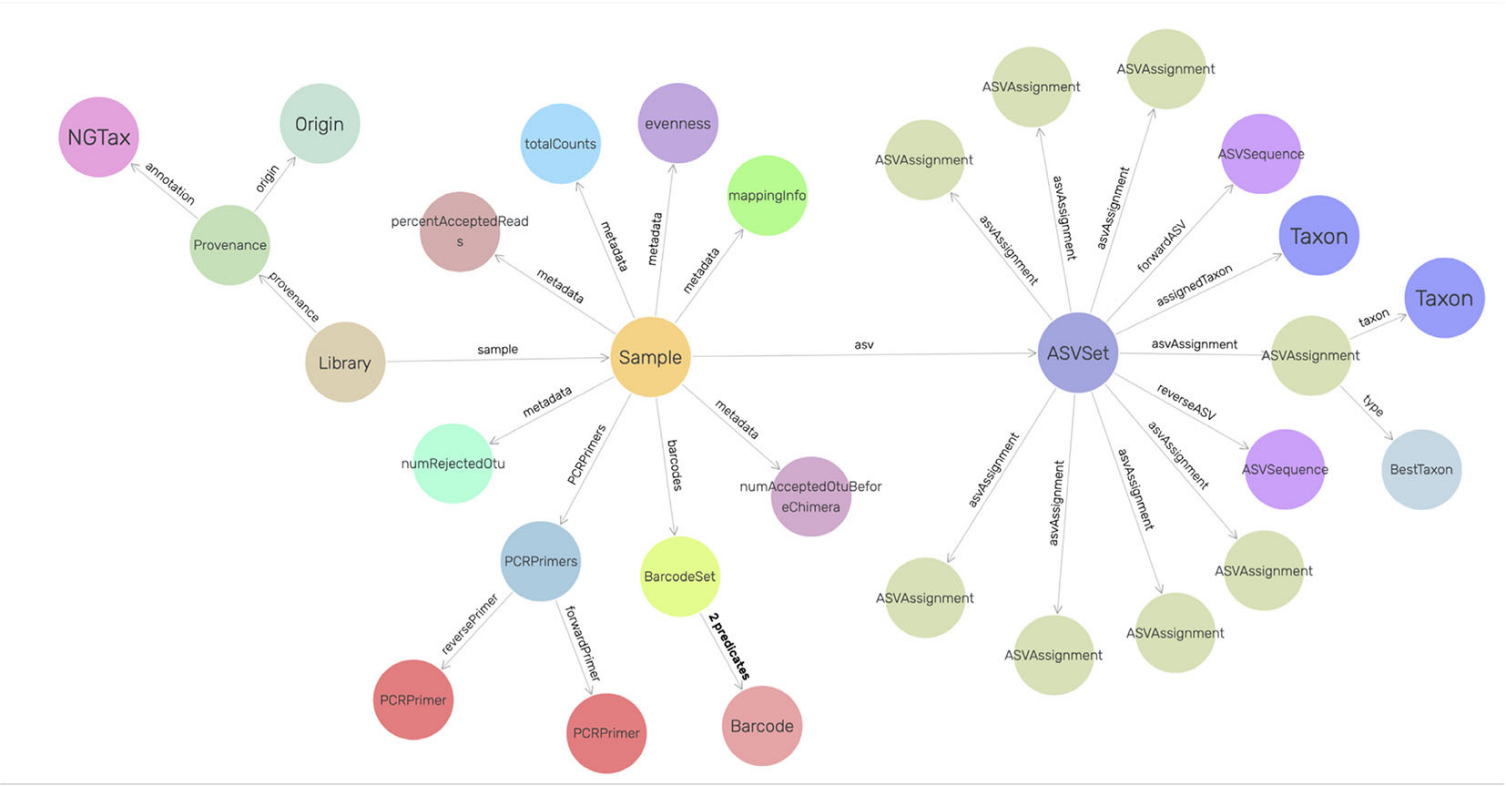
Graphical view of the NG-Tax 2.0 data management model. Nodes are defined in the GBOL ontology. *Sample* and *ASVset* are main hubs and represent sample input and NG-Tax 2.0 processed data. Each *ASVset* represents a specific ASV object, consisting of a collection of (inter)linked descriptions of entities representing data, knowledge and associated meta data of the specific ASV. Each *ASVset* is directly linked to the *Sample* node which is used as a hub for the experimental dependencies. Each *Sample* is part of a *Library* containing information of an individual sequence and analysis run. The visualization was done in GraphDB (http://graphdb.ontotext.com/) using the visual graph interface.

To increase human readability, ontology class names represent the underlying concept as closely as possible. Classes start with uppercase whereas properties start with lowercase. *Library* is the root of the ontology. Each *Library* contains samples according to the input mapping file and it also refers back to the metadata and the command arguments. Each sample contains ASV objects composed of the forward and reverse sequence of the particular ASV, the number of amplicon reads in the sample that have this particular forward and reverse sequence and their taxonomic annotation. The ASVAssignment class is a class where all the possible taxonomic hits of the ASV objects are stored (**Figure 2**). The NG-Tax 2.0 ontology is integrated in the Genome Biology Ontology Language available at http://gbol.life (van Dam et al., 2019).

### ASV-Picking, Artefact Filtering and Correction for the Impact of Error-Reads on the Relative Abundance Estimates

NG-Tax 2.0 can handle both single and paired-end reads. In NG-Tax 2.0 paired-end reads are filtered for matching primers and barcodes but not merged and reads are subsequently processed in parallel. As the forward and reverse read may significantly differ in quality and reverse reads may require additional trimming, in NG-Tax 2.0 the forward and reverse reads are not necessarily of the same length and therefore two parameters are used (-for_read_len and -rev_read_len) to define read lengths used for ASV formation. If the -rev_read_len parameter is not set, single reads or merged forward and reverse reads can be used in the analysis.

NG-Tax 2.0 error-handling is built on the assumption that erroneous reads are more likely to be less abundant than true biological variation. In addition, it is assumed that erroneous sequences (reads with random sequencing errors and (amplified) reads systematic sequence errors) have a high degree of sequence similarity with true reads amplified from the same template sequence in the sample. To deal with such erroneous sequences NG-Tax 2.0 does not start from individual reads or read-pairs but first builds a collection of initial ASV objects from the pool of available reads. In NG-Tax 2.0 by default three (default, user defined) or more identical forward and reverse sequences will form an ASV object and the thus clustered forward and reverse sequences of this object are subsequently used as a reference sequences in the two-step error handling.

NG-Tax 2.0 first assumes that the remaining (singleton) read-pairs are unable to join an already existing ASV object because of a random sequencing error. NG-Tax 2.0 uses a k-bounded Levenshtein function and a cumulative edit distance of one nucleotide (mismatch or indel) to find a match between ASV objects and singleton read pairs. If a singleton ASV read pair shows a single mismatch (mutation or indel) with an ASV reference in either the forward or the reverse read, it is assumed this is due to a random sequence error and the singleton is joined with the particular object thereby increasing the read count of the object but not changing the original sequences linked to the object. Singletons showing more than one mismatch are considered as sample specific noise and discarded.

Secondly, due to PCR and sequence-specific errors (Shin and Park, 2016), specific amplicons may also accumulate above-average sequencing errors resulting in the formation of an erroneous ASV object. Here the assumption is that an erroneous ASV object will show a high degree of sequence similarity with an also existing true ASV object. To find erroneous ASV objects, NG-Tax 2.0 ranks ASV-objects by read counts and uses the k-bounded Levenshtein function to merge ASV objects with read-count below a set threshold, with ASV objects with read counts better than the set threshold starting with the ASV object with the highest read count. If a selected ASV object below the threshold has a single mismatch (mutation or indel) with a high read-count ASV object the two ASV objects are merged. The sequences of the high read-count object are kept because they are believed to be true and the read-counts of both objects are summed. For this merging process a user defined relative abundance threshold is used and by default this is set to 0.1% of the total number of read-pairs associated with ASVs. If NG-Tax 2.0 cannot merge an ASV object with a read count below the set threshold, it will be labelled as ‘provisionally rejected” but the ASV object remains in the output file for further analysis as it could be a true variation, and therefore the first 100 (default, user defined) most abundant provisionally rejected ASVs also obtain a taxonomic assignment. However, most of these flagged ASVs are likely to be sample specific noise (Faith et al., 2013). To show that provisionally rejected ASVs are likely noise we followed their fate in a closed biological system. Samples were obtained from a dietary intervention in an *in vitro* system that simulates the dynamics conditions in the human colon (**Data Sheet 3**). To show reproducibility, several replicates were taken. Because we do not delete but only label as such, sample specific provisionally rejected ASVs we can track their presence over multiple replicates and samples using SPARQL queries. The sequences of almost all provisionally rejected ASVs were only present in a single sample. The percentage of flagged as rejected ASVs that were present in at least two individual samples, ranged from 2.7 to 5.4%, which indeed suggests that the vast majority of the flagged ASVs is likely sample specific noise.

### Taxonomic Assignment of ASV Objects

NG-Tax 2.0 uses reference fasta or alignment files obtained from repositories such as the ARB-SILVA database (Quast et al., 2012) for taxonomic assignments. To reduce the computational load, reference sequences are trimmed such that they include only the region matching the reads. The length of the regions of interest are defined by the length of the reads in the ASV object while the location of the amplicon primer sequences in the reference sequences are used to mark the 5'- and 3'-end of the region of interest. Subsequently, the thus reduced reference file is converted into a look-up table by clustering and counting entries that are identical in sequence and in taxonomic annotation. This look-up table is automatically re-used when different sets of samples with the same parameters are processed. Using the k-bounded Levenshtein function with an upper-bound of 50, the edit distance between each ASV read pair and entries in the reference file is measured. For each edit distance with a maximum sequence mismatch between the reference sequence and the amplicon sequence of 15%, a list of sequence entries, including frequency of occurrence in the reference database file and taxonomic annotation is generated and stored as an integral part of the particular ASV object. This list is also included in the exported extended Biom file. Following a set of rules outlined below, the classifier subsequently proposes from this list of candidates the most likely taxonomic assignment by taking into account the number of mismatches. Depending on the level of sequence identity with the reference set, by default the lowest possible taxonomic ranks proposed by NG-Tax 2.0 will be used, out of species, genus, family and order. Species will only be assigned when a perfect match is obtained with a single species. Between 100-95% sequence identity the lowest proposed taxonomic assignment is genus, between 95-92% the level is family and below 92%, the level is order. These values are stored as attributes of the *CommandArgs* class. Note that while these rules provide a tentative taxonomic assignment based on best practices, for each ASV the full list of reference database sequences remains available and can be retrieved and compared by querying the graph database at any time through a SPARQL endpoint.

### Analysis of NG-Tax 2.0 Precision and Recall Using Mock Communities

**Figure 3:**
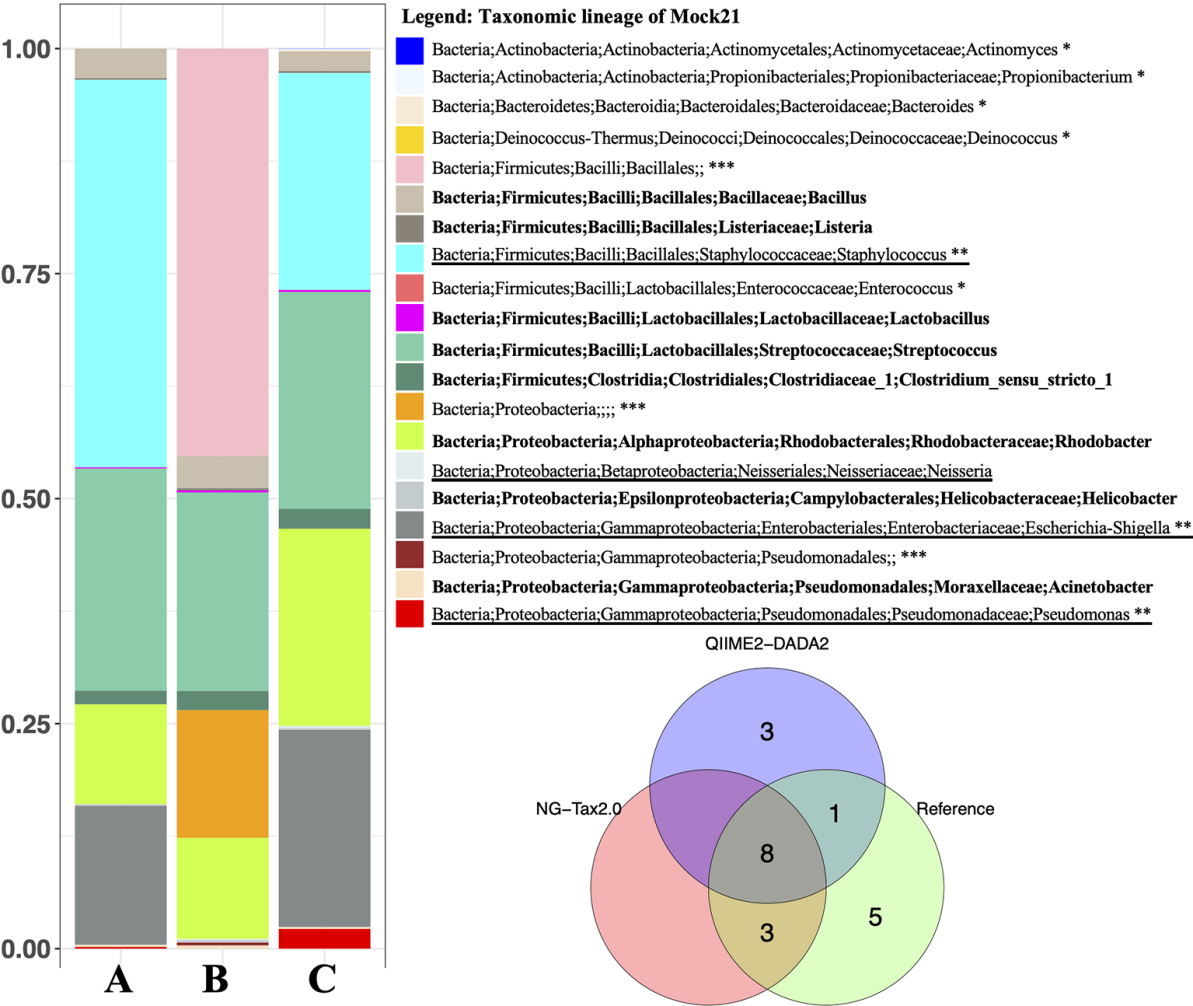
Microbial composition of Mockrobiota mock community 21. Mock21 is a staggered mixture of 20 bacterial strains. *Left* mock21 NG-Tax 2.0 and DADA2 predictions using the ARB-Silva reference database 123 for taxonomic annotation. In this comparison, for each tool the optimal read length was used: 140nt for NG-Tax 2.0 and 220nt for DADA2. **(A)** NG-Tax 2.0. **(B)** DADA2. **(C)** Reference composition. Identical results were obtained with the reference database with 97% and 99% similarity thresholds. In bold, mock21 reference taxons correctly identified with NG-Tax 2.0 and DADA2. *Mock21 reference taxons not detected by either tool, **Mock21 reference taxons detected by NG-Tax 2.0 but not DADA2. *Underlined*, mock 21 reference taxons detected by DADA2 but not NG-Tax 2.0. ***No prediction at genus level, however correctly assigned the taxonomic lineage. *Right* Venn diagram summarizing the taxonomic annotation results.

We measured NG-Tax 2.0 precision and recall using ten staggered and evenly distributed MiSeq 16S rRNA gene mock communities (**Table 1**) obtained from the Mockrobiota public repository. Then communities were analysed in parallel with the DADA2 implementation in the QIIME2 pipeline (from here on referred to as DADA2). NG-tax 2.0 and DADA2 taxonomic predictions were compared using different read lengths and three consecutive stable versions of the ARB-SILVA reference database. The reference composition of the selected mock communities is based on SILVA version 123 using a similarity threshold of 97% and 99% respectively. **Figure 3** displays a typical example showing a compositional analysis of mock21 using either NG-Tax 2.0 or DADA2. For each tool, the optimal read length was used.

**Figure 4:**
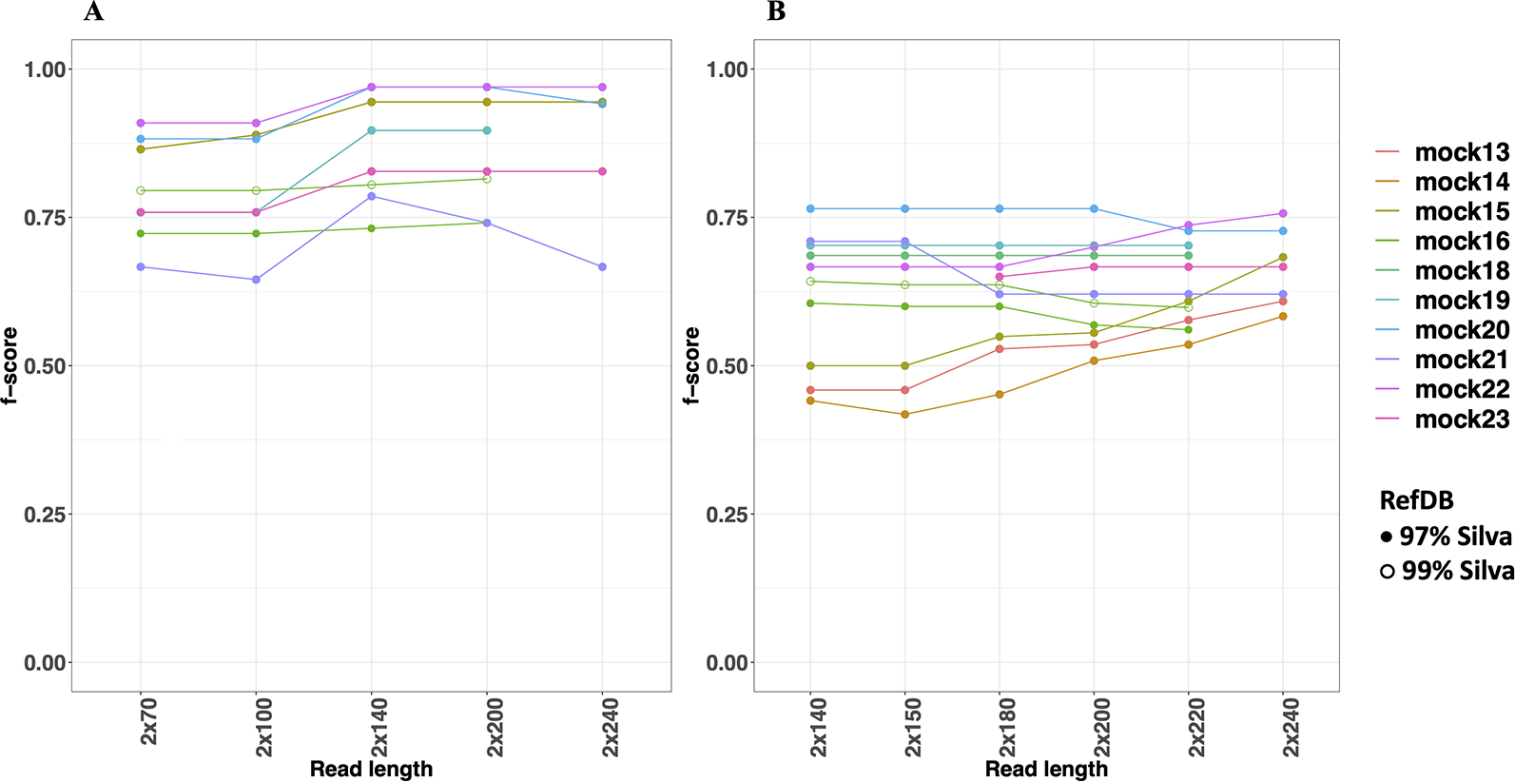
F-scores of NG-Tax 2.0 **(A)** and DADA2 **(B)** at different read length. Silva 123 was used as reference database. The *x*-axis indicates the trimmed read length of the forward and reverse read. Note that mock16, mock 18 and mock 19 were not included in the comparison of the 240nt read length as after removal of primer sequences these reads were too short.

The metrics used to compare and evaluate the performances of both pipelines were recall, precision and F-score. F-score is a single metric that combines both recall and precision and is used here to select an optimal read length for the analysis. When considering F-scores from both pipelines for different mock communities at different read lengths, NG-Tax 2.0 had a higher range of 0.65 to 0.97, compared to DADA2’s 0.42 to 0.76, across all mock communities (**Figure 4**). Moreover, NG-Tax 2.0 revealed an optimal read length at 140 nucleotides with F-scores ranging from 0.73 to 0.97 across all the communities. In contrast, DADA2’s optimal read length varied between mock communities, which suggests that the performance of this tool in this respect may depend on the sample composition. We therefore selected a fixed read length per tool for further analysis: 140nt for NG-Tax 2.0 and 220nt for DADA2 as they provide the highest mean of the F-score calculated from all the communities at that length, which results in 0.89 and 0.64 respectively.

**Figure 5:**
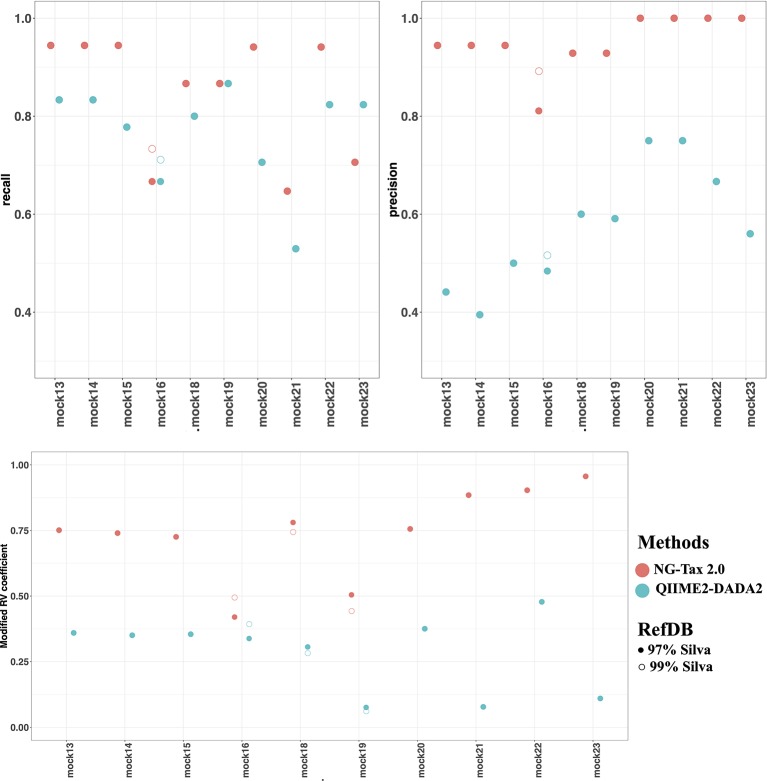
Recall, precision and modified RV coefficient of NG-Tax 2.0 and DADA2. NG-Tax 2.0 is labelled in *red* and DADA2 is labelled in *blue*. *Upper panel* left, recall; right, precision. *Lower panel* modified RV coefficient. Silva 123 is used as reference database clustered at 97 (*filled circles*) and 99% (*open circles*). Note that in many cases results overlap in which case only the results obtained with the 97% threshold is shown.

The two factors that contribute to the F-score are recall and precision. Both can be used to assess the quality of the pipeline and are equally important. In general, the level of recall of DADA2 and NG-Tax 2.0 were comparable with an average of 0.77 and 0.85, respectively. However, the precision of NG-Tax 2.0 was noticeably higher than that of DADA2 with an average of 0.95 vs 0.58 (**Figure 5**). The results show that both tools are equally good at inferring the expected microbial composition from the sample. However, DADA2 tended to predict taxonomic assignment of a higher rank, which led to a lower precision and F-score. Similar results using two staggered mocks from Tourlousse et al., 2017 with two replicates each can be found in **Data Sheet 3**.

### Modified Rv Coefficient

An alternative metric used to determine the efficiency of both pipelines is the modified RV coefficient. Unlike the previous statistical measures, the modified RV coefficient takes into account the relative abundance of the identified bacteria, which is crucial for understanding a pipeline’s performance. **Figure 5** shows that the modified RV coefficient from NG-Tax 2.0 on both the number of taxonomic lineages and their corresponding relative abundances are closer to the actual composition than DADA2. The average for NG-Tax 2.0 is 0.74 whereas the average coefficient for DADA2 is 0.28.

### Tracking of Asvs Across Multiple Samples

**Figure 6:**
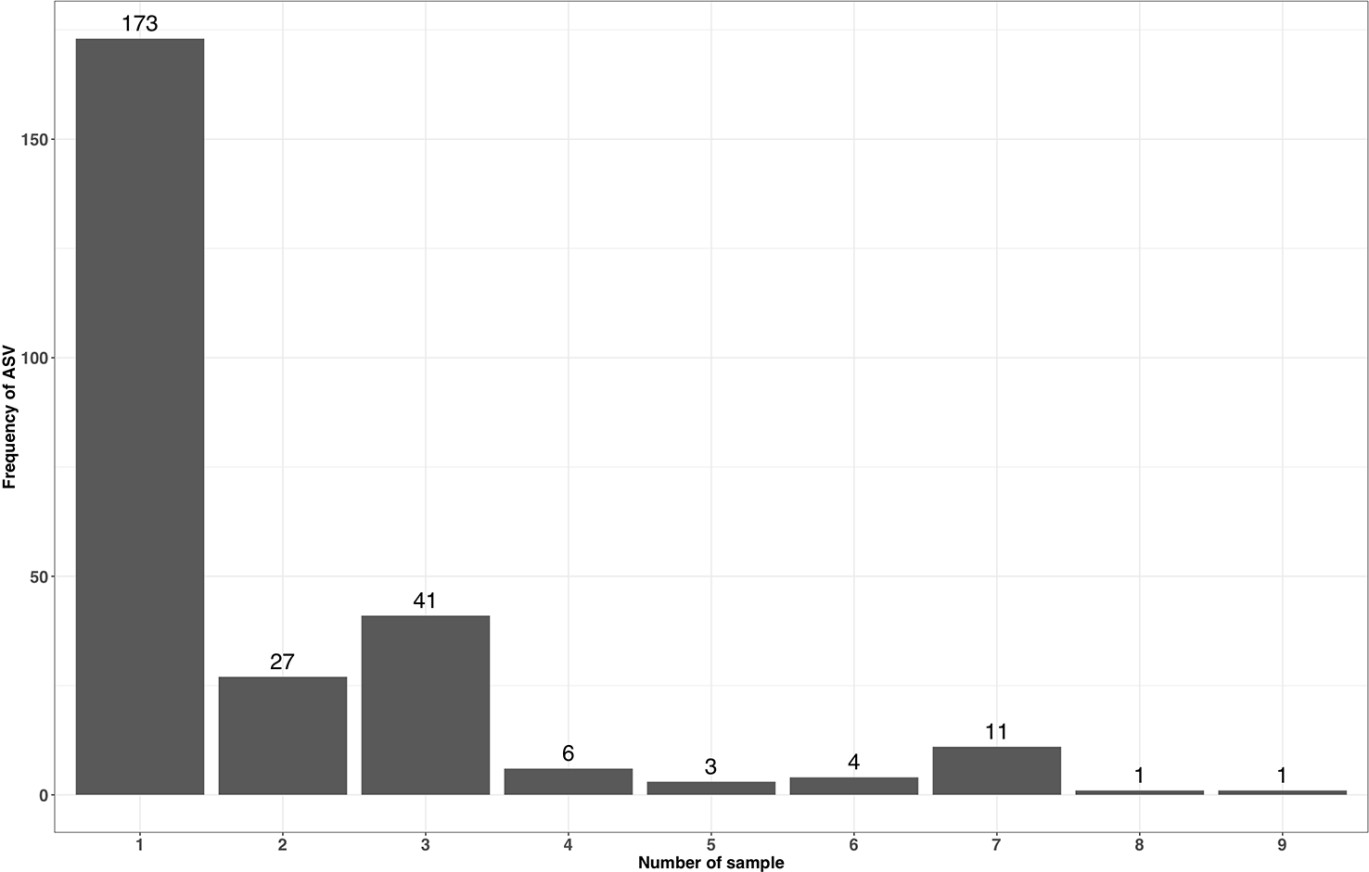
Occurrence of accepted ASV forward and reverse sequences with a read length of 70 across multiple mock samples.

ASVs have a single nucleotide resolution and are assumed to be directly derived from an existing biological entity. As in NG-Tax, ASV objects contain the forward and reverse sequence of the specific ASV (**Figure 2**), we can design SPARQL queries to explore the presence of specific ASVs across multiple mock samples. As most of the selected mocks are not biologically related, the majority of the ASVs will only be present in a single sample. Mock13-15, however, are composed of genomic DNA from the same 21 bacterial isolates and thus we expected a high number of ASVs shared between these three samples. The composition of mock13-15 includes three *Streptococcus* species being *Streptococcus agalactiae* ATCC BAA-611, *Streptococcus mutans* ATCC 700610, and *Streptococcus pneumoniae* ATCC BAA-334, each of which has multiple, but not necessarily identical copies of the 16S rRNA gene. For instance, the *Streptococcus agalactiae* genome contains seven copies of the 16S rRNA gene. Nine distinctive mock13 ASV objects are taxonomically annotated as *Streptococcu*s and amplicon sequences linked to five of those objects showed 100% sequence identity with separate *Streptococcus agalactiae* 16S rRNA genes. A SPARQL query showed that four of these ASVs are present in all three mocks while one is not present in mock14. Overall, of the 60 taxonomically annotated ASVs in mock13, 56 variant sequences are present in all three mocks. Similarly, when we include in the query the unrelated mock16 composed of genomic DNA from 57 bacterial isolates, the expected taxon overlap is four; *Bacteroides*, *Porphyromonas, Deinococcus* and *Enterococcus*. The SPARQL query showed that five distinct ASVs are present in all four mocks. Two ASV’s were annotated as *Bacteroides*, the other three as *Porphyromonas, Deinococcus* and *Enterococcus*. **Figure 6** summarizes the result of a SPARQL query for the presence of specific ASVs amplicon sequence variants across all mocks.

### Impact of Incremental Databases

The taxonomic annotation of a 16S rRNA gene amplicon depends on many variables, including the version of the reference database used. Because new phylogenetic groups are constantly being discovered (Hug et al., 2016), obtaining a correct bacterial phylogeny will remain a moving target for some time. Hence, keeping track of how the amplicon data was analysed, the data provenance, is critical. The observation that even a single reference database, clustered at two different similarity thresholds can lead to different results led us to investigate the impact of incremental versions of the SILVA database. For this, we used SPARQL queries to analyse the taxonomic annotation of the ten selected mock communities using three incremental stable versions of the SILVA database, namely releases 123, 128 and 132. NG-Tax 2.0 has the ability to create a custom taxonomic reference file *de novo* using a set of unaligned reference sequences as input. This allows for instance to add a new species to an existing taxonomic reference file. To demonstrate this feature we built a custom reference file using 16S rRNA gene sequences obtained from Hug et al., 2016. The SILVA result showed that in the latest version of the SILVA database some taxa have been reclassified. For instance, in mock18 the phylum and class of *Treponema_2* have been reclassified from *Spirochaetae* and *Spirochaetes* to *Spirochaetes and Spirochaetia*. The class and order of *Nitrosomonas* were also reclassified from Betaproteobacteria and *Nitrosomonadales* to Gammaproteobacteria and *Betaproteobacteriales*. Not unexpected the biggest “change” was when we compared taxonomic reference files from different origins. Results are summarized in **Table S1**.

## Discussion

NG-Tax 2.0 is an open software framework that uses semantic technologies for data and knowledge management. It is particularly designed for FAIR and high-throughput taxonomic classification and downstream analysis of marker gene amplicon sequences. By using the RDF data model, NG-Tax 2.0 is able to engage a traceable *de novo* OTU picking and de-noising algorithm, generating ASV objects that link ASV sequence data with the full data-wise and element-wise provenance. The linked data structure ensures a high degree of interoperability. Serialized ASV objects can be automatically stored in a standard graph database structure and directly queried for comparative analyses of data and meta-data across thousands of samples.

For targeted amplicon sequencing, denoising, i.e. the separation of biological variation from amplicon sequencing errors, is essential to increase the reliability of downstream analyses. Clustering sequences into OTUs has been routinely applied in the past to reduce the impact of sequence errors and to speed up the analysis process by picking a representative sequence (Nguyen et al., 2016). However, many recent studies now use a 100% similarity threshold or ASVs. ASVs are standardly generated with NG-Tax 2.0 and with DADA2, one of the most commonly used pipelines today. As both NG-Tax 2.0 and DADA2 have a single nucleotide resolution, the number of ASVs and taxonomic annotation from NG-Tax 2.0 and DADA2 should be the same, however, the specific criteria used to remove erroneous-sequences creates the differences.

To test the performance of NG-Tax 2.0 we used ten 16S rRNA gene mock communities, staggered and even, and compared the results with those obtained with DADA2. We showed that while the recall of the expected microbial composition for both pipelines was comparable, there are substantial differences in the precision and the prediction of relative abundances. We proposed the use of a modified RV coefficient for evaluating the performance of a given pipeline (Smilde et al., 2009). It measures the common information of two matrixes which represent the relative abundance distributions of the microbial composition. This increases the efficiency in differentiating between two communities as compared to the binary classifier. The advantage in using this method is the ease of interpretation. The results are presented as a single value, which is convenient for visualization, and it can be interpreted in the same way as a correlation coefficient with the value between -1 and 1, which is already familiar to biologists.

Discussions about how to analyse microbial community data is an on-going process, and the golden standard for microbiome analysis has not yet been settled (Knight et al., 2018; Pollock et al., 2018). DADA2 generates a parametric error model based on the dataset and uses it to remove or collapse the sequences. On the other hand, NG-Tax 2.0 employs an empirically determined relative abundance cut-off taking into account the evenness of the read distribution over the ASVs to flag ASVs with an associated low read count that are considered as artefacts. It then attempts to merge those artefacts with ASVs with high read counts, which are more likely to be true ASVs, using a single mismatch as criterium. While both methods seem to be effective in recalling the expected composition, precision of NG-Tax 2.0 was much higher than that of DADA2 mainly because the parametric model predicted more ASVs, an effect that will increase along with the diversity of the community (Nearing et al., 2018).

NG-Tax 2.0’s novelty is in using the RDF data model to transform amplicon data into ASV objects that link ASV sequences data with the dataset-wise and element-wise provenance. This not only greatly enhances the reproducibility of the analysis but also increases the degree of interoperability of the data required for comparative analyses. For instance, in finding rare species in a particular community, DADA2 may have the advantage while at the same time risking that those organisms are artefacts. In NG-Tax 2.0, rejected ASVs with relatively low read abundances are flagged as artefacts but due to a high degree of interoperability NG-Tax 2.0 enables a reanalysis of the data by comparing them between multiple samples and by using alternative parameter settings.

To conclude, NG-Tax 2.0 provides a simple to use, semantic framework for high-throughput microbiota analysis. Due to use of the RDF data model it allows to generate fully traceable ASV objects that link ASV sequence data with the full data-wise and element-wise provenance. This data model allows users to systematically adjust the parameters for the reanalysis or infer the biology behind these sequences using comparative analyses.

We compared the analysis results from the publicly available mock communities against those obtained by DADA2. The outcome shows that both pipelines are able to recall the microbial composition from the reference. However, NG-Tax 2.0 shows a higher precision score and the predicted relative abundances are closer to the expected composition than those provided by DADA2.

## Data Availability Statement

All datasets generated for this study are included in the article/**Supplementary Material**.

## Author Contributions

JD, WP, PS, and JK developed the code. WP, PS, and JK performed the computational analyses. WP and GH wrote the original draft of the manuscript. WP, GH, JD, JK, HS, and PS contributed to the writing, review, and editing of the manuscript.

## Funding

WP is financially supported by a Royal Thai Government Scholarship, Thailand. This work has received funding from the Netherlands Organisation for Scientific Research funded UNLOCK project (NRGWI.obrug.2018.005) and from the European Union H2020 under grant agreement No. 730976 (IBISBA 1.0).

## Conflict of Interest

The authors declare that the research was conducted in the absence of any commercial or financial relationships that could be construed as a potential conflict of interest.
