# Supplementary material for: NG-Tax 2.0: A Semantic Framework for High-Throughput Amplicon Analysis
Source: Front Genet. 2020 Jan 23;10:1366. doi: 10.3389/fgene.2019.01366 (PMC6989550; doi:10.3389/fgene.2019.01366)
Supplement: Supplementary file 1 [file DataSheet_1.pdf]

Supplementary file S1: example SPARQL queries

NG-Tax 2.0: A Semantic Framework for High-throughput Amplicon Analysis.

Poncheewin W[1,#], Hermes G. D. A.[2,#], van Dam J.C.J[1], Koehorst J.J[1], Smidt H[2], Schaap P.J.[1*]

1 OTU table

```
PREFIX gbol:  <http://gbol.life/0.1/>

select ?sample ?clusteredReadCount ?taxonName

where {

   ?lib a gbol:Library .

   ?lib gbol:sample ?sample .

   ?sample gbol:name ?name .

   ?sample gbol:asv ?asv .

   ?asv a gbol:ASVSet .

   ?asv gbol:assignedTaxon ?assignedTaxon .

   ?asv gbol:clusteredReadCount ?clusteredReadCount .

   ?assignedTaxon gbol:taxonName ?taxonName .

}
```

2 ASV assignment comparison between databases

```
PREFIX gbol: <http://gbol.life/0.1/>

SELECT DISTINCT ?fseq ?rseq ?taxName7 ?dbName ?mismatchDB ?sampleName

WHERE {

  ?library gbol:provenance ?prov .

  ?prov gbol:annotation ?annot .

  ?annot gbol:refdb ?db .

  bind (replace (?db , "../database/", "") AS ?dbName)

  ?annot gbol:mismatchDB ?mismatchDB .

  ?library gbol:sample ?sample .

  ?sample gbol:name ?sampleName .

  ?sample gbol:asv ?asv .

  ?asv a gbol:ASVSet .

  ?asv gbol:forwardASV ?fasv .

  ?fasv gbol:sequence ?fseq .

  ?asv gbol:reverseASV ?rasv .

  ?rasv gbol:sequence ?rseq .

  ?asv gbol:assignedTaxon ?tax .

  ?tax gbol:taxonName ?taxName .

  bind (replace (?taxName , "d__", "") AS ?taxName2)

  bind (replace (?taxName2 , "p__", "") AS ?taxName3)

  bind (replace (?taxName3 , "c__", "") AS ?taxName4)

  bind (replace (?taxName4 , "o__", "") AS ?taxName5)

  bind (replace (?taxName5 , "f__", "") AS ?taxName6)

  bind (replace (?taxName6 , "g__", "") AS ?taxName7)

} ORDER BY ?fseq ?rseq
```

3 Tracing the occurrence of the same ASVs in different samples

```
PREFIX gbol: <http://gbol.life/0.1/>

PREFIX xsd: <http://www.w3.org/2001/XMLSchema#>

SELECT DISTINCT ?fseq ?rseq (COUNT (DISTINCT ?sampleName) AS ?count)

WHERE {

  ?library gbol:provenance ?prov .

  ?prov gbol:annotation ?annot .

  ?annot gbol:refdb ?db .

  bind (replace (?db , "../database/", "") AS ?dbName) .

  FILTER regex(?dbName, "SILVA_132_SSURef_tax_silva.fasta.gz") .

  ?annot gbol:mismatchDB ?mismatchDB .

  FILTER (?mismatchDB = "true"^^xsd:boolean) .

  ?library gbol:sample ?sample .

  ?sample gbol:name ?sampleName .

  ?sample gbol:asv ?asv .

  ?asv a gbol:ASVSet .

  ?asv gbol:forwardASV ?fasv .

  ?fasv gbol:sequence ?fseq .

  ?asv gbol:reverseASV ?rasv .

  ?rasv gbol:sequence ?rseq .

} GROUP BY ?fseq ?rseq
```

More example queries can be found: http://wurssb.gitlab.io/ngtax/
