# Supplementary material for: NG-Tax 2.0: A Semantic Framework for High-Throughput Amplicon Analysis
Source: Front Genet. 2020 Jan 23;10:1366. doi: 10.3389/fgene.2019.01366 (PMC6989550; doi:10.3389/fgene.2019.01366)
Supplement: Supplementary file 3 [file DataSheet_3.pdf]

Supplementary file S3: Additional analysis of provisionally rejected ASVs and accuracy assessment of an additional staggered mock community.

NG-Tax 2.0: A Semantic Framework for High-throughput Amplicon Analysis.

Poncheewin W[1,#], Hermes G. D. A.[2,#], van Dam J.C.J[1.], Koehorst J.J[1], Smidt H[2], Schaap P.J.[1*]

1. Analysis of the occurrence of provisionally rejected ASVs in multiple samples

The vast majority of the 'flagged as rejected' ASVs are probably noise (see also Faith et al, 2016 Fig 1B) and only when the researcher has good confidence that such an ASV represents a 'real species' it should be retrieved. To exemplify the noise levels below the 0.1% threshold in more complex biological samples and how these can be retrieved we have added an experimental data set.
The samples are obtained from a dietary intervention in an *in vitro* system that simulates the dynamics conditions in the human colon: TIM-2 (https://en.wikipedia.org/wiki/TNO_intestinal_model).

The system was inoculated with the pooled microbiota of seven healthy individuals, which has been incubated in a fed-batch fermentor, before inoculation of the system. The samples were from a single fermentation run at t=0,24,48 and 72h. To show reproducibility, sample 1 consists of a sequencing duplicate (same sample sequenced twice on different occasions; sample 1A and sample 1B) and a PCR duplicate (same sample with a different barcode; sample 1C*). The TIM2 system is mainly a closed system and therefore we expected the number of ASVs to remain relatively stable during a fermentation run. Because the flagged rejected ASVs of individual samples are not deleted but only labeled as such we can track them over multiple samples using the Semantic framework in conjunction with a SPARQL query. Results are shown in the table. An example SPARQL query is provided below.

Sample	Reads	Flagged ASVs	Accepted >=2 (%)	Accepted >=3 (%)
1A	211564	1206 (74%)	5.4	1.7
1B	457274	2215 (81%)	3.5	1.1
1C*	389840	1849 (76%)	2.7	0.7
2	135271	750 (70%)	4.1	3.3
3	304383	1577 (79%)	4.2	1.5
4	309674	1946 (81%)	3.2	1.8

The percentage of flagged as rejected ASV that were present in at least two individual samples, ranged from 2.7-5.4%, which suggests that the vast majority of the flagged ASVs is likely sample specific noise.

1. Faith, J.J., et al., *The long-term stability of the human gut microbiota.* Science, 2013. **341**(6141): p. 1237439.

2. SPARQL query used to obtain the sample name, ASVs and ASV type

```
PREFIX gbol:<http://gbol.life/0.1/>
SELECT ?name ?type ?fasv ?rasv
WHERE {
   ?sample a gbol:Sample .
   ?sample gbol:name ?name .
   ?sample gbol:asv ?asv .
   ?asv a ?type .
   ?asv gbol:forwardASV ?fasv .
   ?asv gbol:reverseASV ?rasv .
}
```

3. Analysis of two additional staggered mock community from Tourlousse et al.

We performed two additional analyses on staggered Mocks from Tourlousse et al. These samples were sequenced using a Miseq machine with 2x250nt in duplicate (rep1 & rep2) with two primers covering V1-V2 (008F – 355R) and V4 (515F – 806R). This supplementary analysis consists of:

1. A comparison of the accuracy of both pipelines
2. The retention of reads for each filtering and/or denoising step
3. A comparison of the effectiveness of denoising using DADA2 and OTU picking in NG-Tax

1. Accuracy

To determine the accuracy of both pipelines, we contrasted NG-Tax with different read lengths (70, 150 and 230nt) with DADA2 using the maximal read length. 230nt for NG-Tax represents the same maximum read without the primer. The taxonomic profiles are shown in Fig 1. F score was comparable for the two methods. Trends for recall and precision are similar to the Mocks in the manuscript. With regards to precision NG-Tax outperformed DADA2 with all read lengths. NG-Tax scores higher on the modified RV coefficient, using 230nt reads and for all 150nt reads except for 515F-806R Rep 2 (Fig 2). Figure 3 shows the quality of the predictions at various taxonomic resolutions for sample 515F806R_rep1.

The various accuracy indicators show similar trends according to the core analysis provided in the main article: using longer read length generally increases the accuracy of taxonomic assignment, However, mainly for V1-V2, 150nt reads resulted in a higher F-score, modified RV coefficient, recall and a similar precision compared to 230nt.

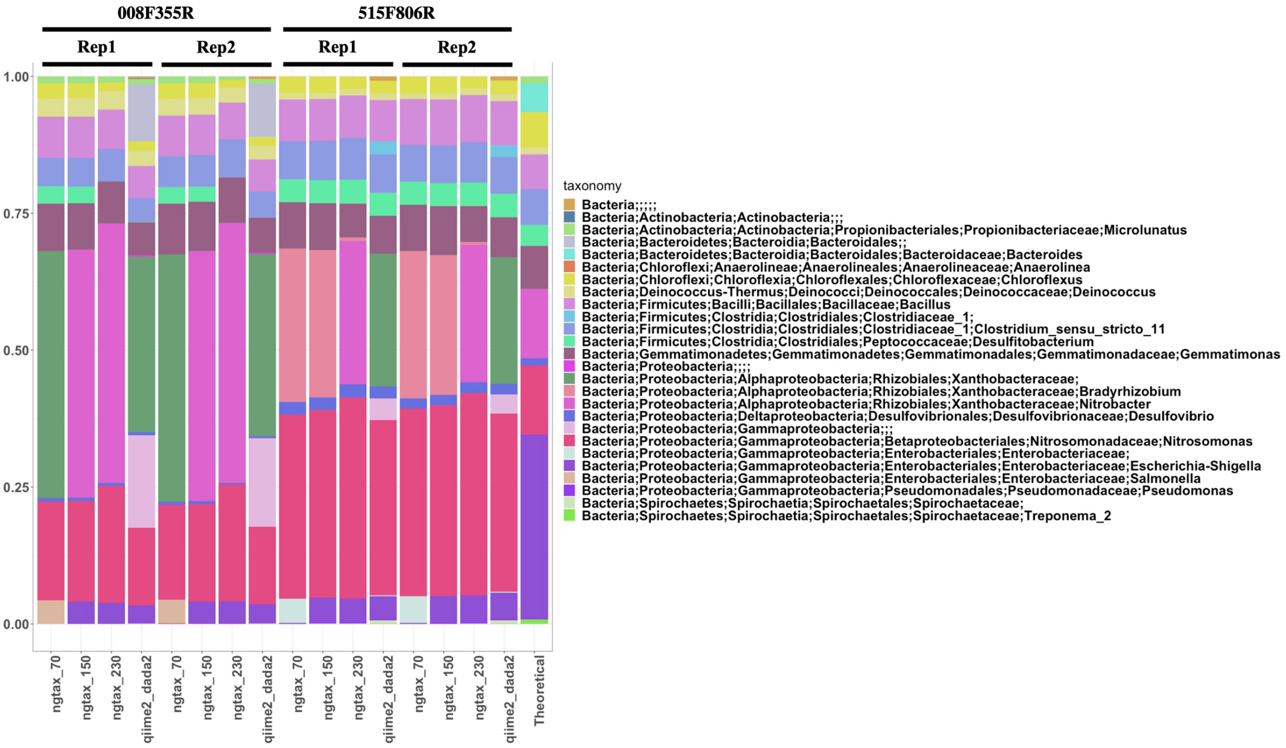

Fig 1. Taxonomic profiles of the sequenced results compared to the reference for replicate 1 and 2 sequenced with primer pairs 008F-335R and 515F-806R covering V1-V2 and V4 respectively.

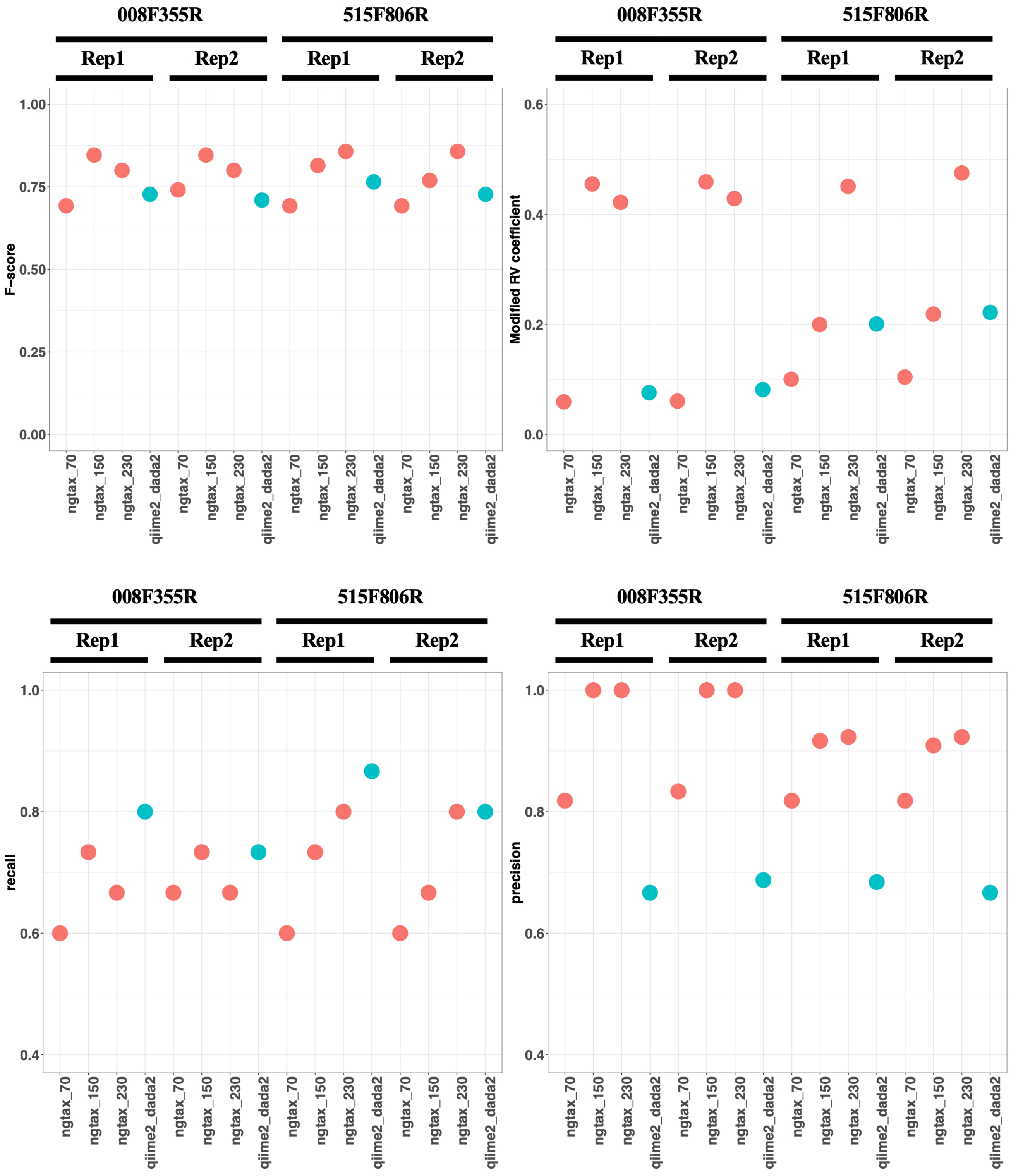

Fig 2. F-scores, modified RV coefficient, recall and precision and of NG-Tax 2.0 and DADA2. NG-Tax 2.0 is labelled in red and DADA2 is labelled in blue.

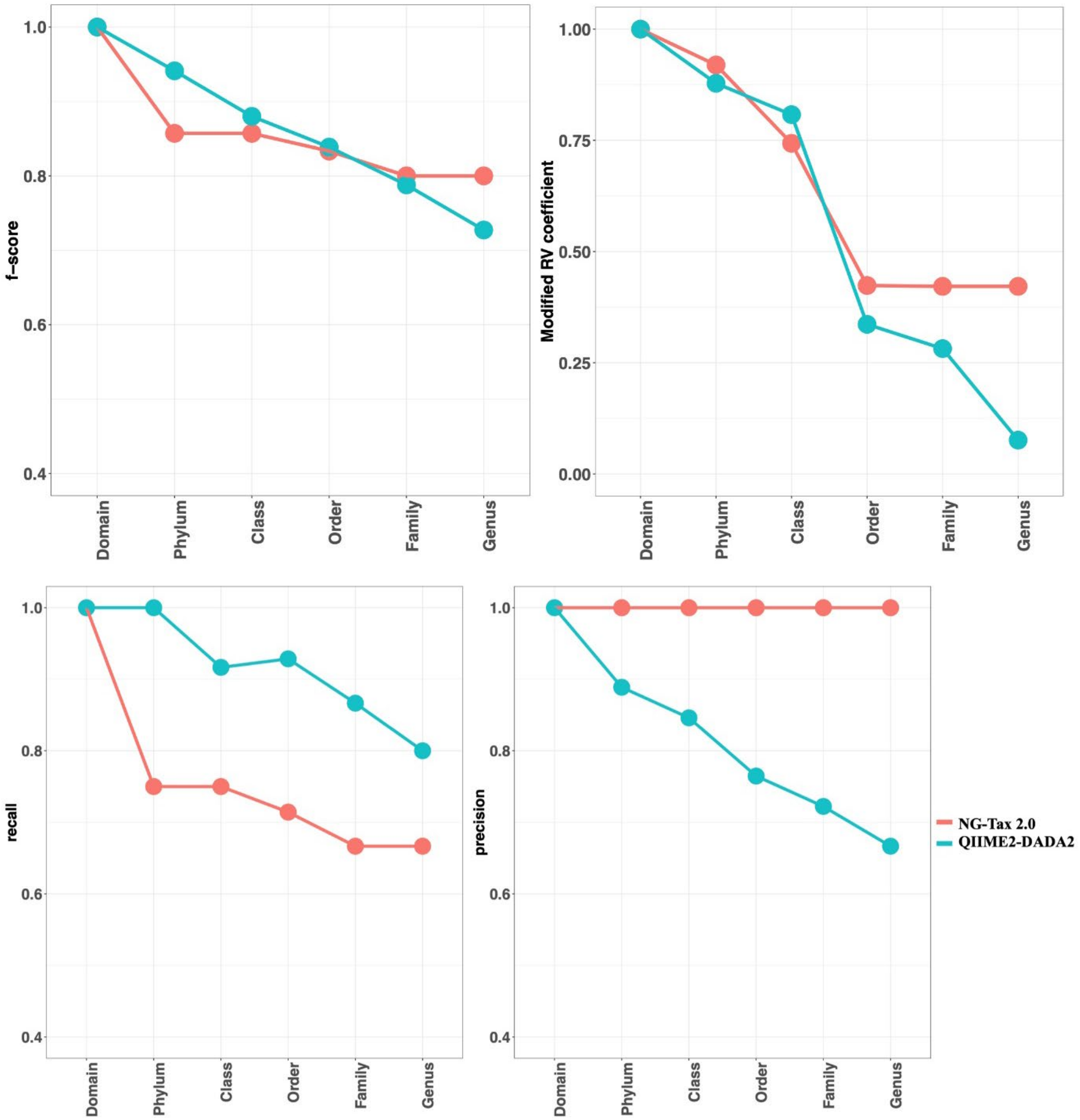

Fig 3. F-scores, modified RV coefficient, recall and precision of NG-Tax 2.0 and DADA2 at different taxonomic resolutions for 515F806R_rep1. NG-Tax 2.0 is labelled in red and DADA2 is labelled in blue.

2. Read retention at each intermediate step for both pipelines

Table 1-4. Number of reads retained after filtering and error correction in NG-Tax 2.0 and after the denoising function of DADA2.

Sample: 515F806R

NG-Tax 2.0													
	Input reads	70				150				230			
		primer match	True ASVs	Chimera	Error Corr	primer match	True ASVs	Chimera	Error Corr	primer match	True ASVs	Chimera	Error Corr
rep1	52283	45092	34070	0	40013	45091	20994	0	27299	44626	13657	0	17541
rep2	26400	22162	16744	37	19683	22162	10383	0	13418	21941	6826	0	8856

QIIME2 – DADA2 (denoising-stats.qzv)					
	Input reads	Filtered	Denoised	Merged	Non-chimeric
rep1	52283	26872	26711	26149	25386
rep2	26400	13546	13426	13109	12843

Sample: 008F355R

NG-Tax 2.0													
	Input reads	70				150				230			
		primer match	True ASVs	Chimera	Error Corr	primer match	True ASVs	Chimera	Error Corr	primer match	True ASVs	Chimera	Error Corr
rep1	35077	23935	18707	0	21859	23935	15517	0	19965	23211	8960	0	13021
rep2	20870	14370	11145	0	13030	14370	9032	0	11708	13951	4831	0	7232

QIIME2 – DADA2 (denoising-stats.qzv)					
	Input reads	Filtered	Denoised	Merged	Non-chimeric
rep1	35077	22886	22806	22756	22306
rep2	20870	12765	12713	12647	12497

3. Comparing the effectiveness of denoising using DADA2 and ~~OTU~~ picking in NG-Tax

To examine the accuracy of unmerged reads for taxonomic microbiota profiling we used sample 515F806R_rep1 as an example. We used DADA2 to denoise the 250nt reads after which the reads were merged using "justConcatenate". From this set forward and reverse reads were extracted to create two FASTA files. These were used as input for NG-Tax 2.0. These results were compared to standard NG-Tax 2.0 using 230nt reads (primers excluded), QIIME2 taxonomic classification using full length reads and the reference.

Denoising with DADA2 produces a similar taxonomic profile as using NG-Tax 2.0 alone, demonstrates that both algorithms produce highly similar results (Fig 4). The modified RV coefficient of DADA2, using NG-Tax 2.0 as a reference is 0.998. Compared to denoising and subsequent classification using QIIME2, both methods score higher in all accuracy metrics (Fig 5).

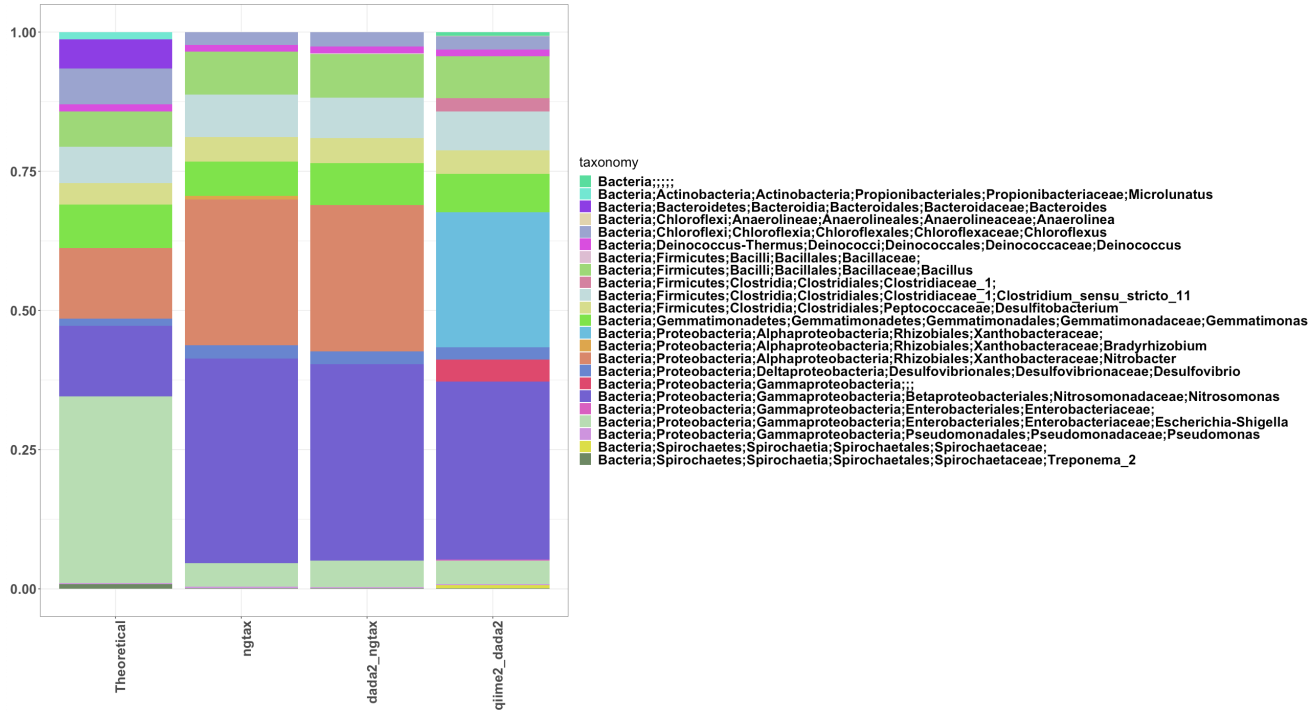

Fig 4. Taxonomic profiles of the sequenced results compared to the reference for sample 515F806R_rep1. Sequenced results are based on NG-Tax, reads denoised with DADA2 used as input for NG-Tax and taxonomic classification with QIIME2 using DADA2 denoised reads.

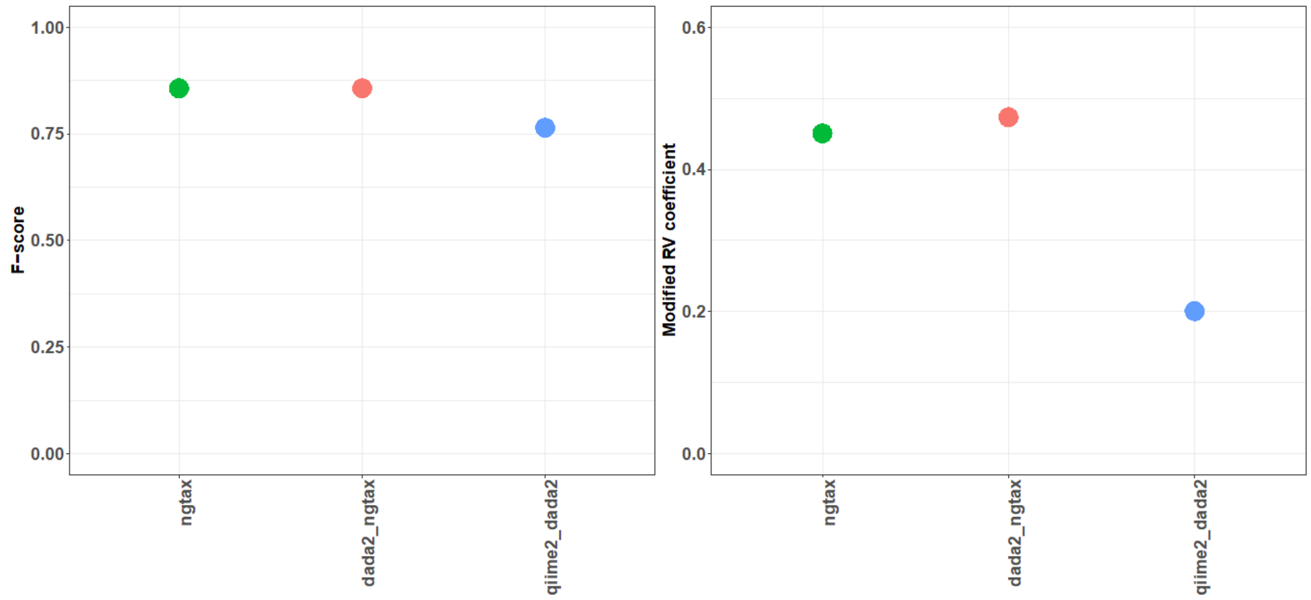

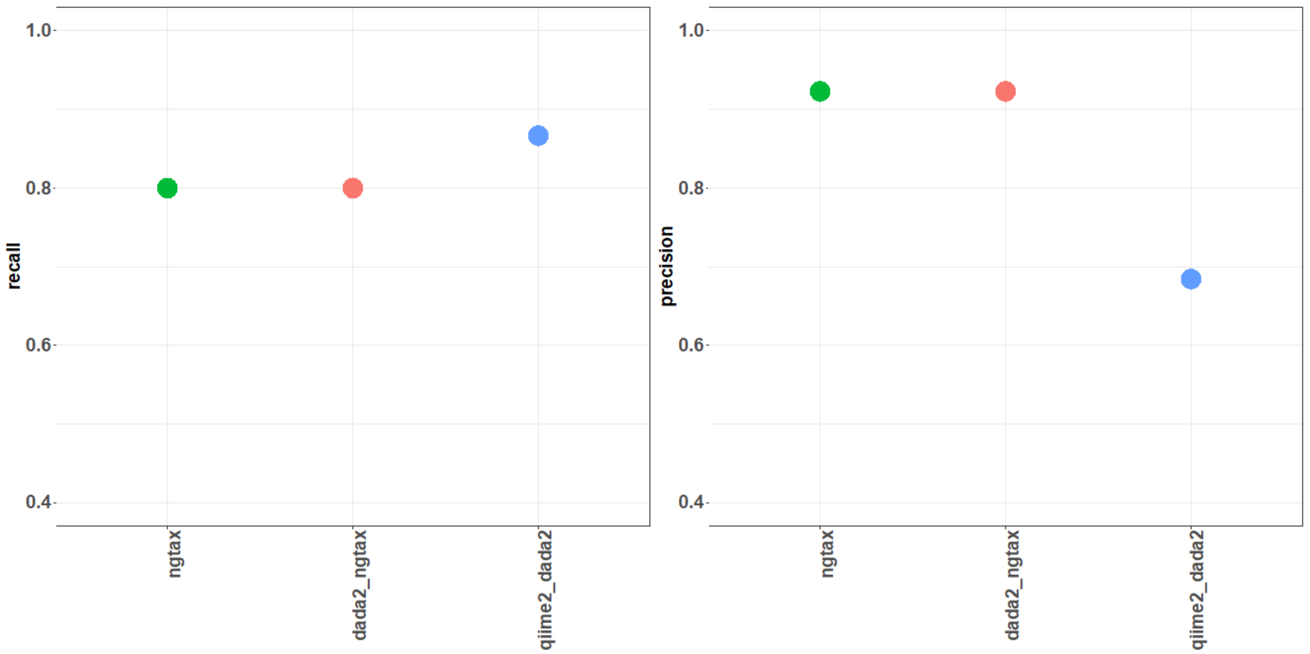

Fig 5. F-scores, modified RV coefficient, recall and precision and of NG-Tax 2.0, DADA2-NG-Tax 2.0 and QIIME2-DADA2.
